# Advanced risk signature analysis of inflammation markers in predicting prostate cancer using the Swedish Apolipoprotein-related MOrtality RISk (AMORIS) cohort

**DOI:** 10.1016/j.esmorw.2025.100156

**Published:** 2025-06-03

**Authors:** G. George, M. Rowley, A.C.C. Coolen, A. Santa Olalla, N. Hammar, M. Feychting, S.N. Karagiannis, D. Enting, M. Van Hemelrijck

**Affiliations:** 1 Transforming Cancer Outcomes Through Research, King’s College London, London, UK; 2 Saddle Point Science Ltd., York, UK; 3 Saddle Point Science Europe, Nijmegen, The Netherlands; 4 Faculty of Science, Radboud University, Nijmegen, The Netherlands; 5 Institute of Environmental Medicine, Karolinska Institutet, Stockholm, Sweden; 6 Cancer Antibody Discovery & Immunotherapy Group, St. John’s Institute of Dermatology, School of Basic & Medical Biosciences & King’s Health Partners Centre for Translational Medicine, King’s College London, London, UK; 7 Breast Cancer Now Research Unit, School of Cancer & Pharmaceutical Sciences, King’s College London, Innovation Hub, Guy’s Hospital, London, UK; 8 Medical Oncology, Guy’s and St Thomas’ NHS Foundation Trust, London, UK

**Keywords:** prostate cancer risk, inflammation markers, signature analysis, AMORIS

## Abstract

**Background:**

Elevated post-diagnosis levels of C-reactive protein (CRP) and haptoglobin, and low albumin levels, have been associated with poor prostate cancer (PCa) prognosis. Advanced techniques are needed for biomarker-based cancer risk prediction. We evaluated PCa risk using Cox models and risk signature analysis in the Swedish Apolipoprotein-related MOrtality RISk (AMORIS) cohort.

**Materials and methods:**

AMORIS includes biomarker data on >800 000 individuals from 1985 to 1996 in primary care and occupational setting, linked to national health and population registers through 2020. PCa risk was analysed using Cox proportional hazard models for albumin, CRP, haptoglobin and white blood cells at the third time point, adjusting for age, socioeconomic status, education level, Charlson comorbidity index (CCI) and cancer history, and risk signature analysis with training and validation sets (preventing overfitting) including repeated biomarker measurements, covariate interactions and baseline factors. Sensitivity analysis categorised age and CCI.

**Results:**

Cox model showed elevated CRP and CCI ≥2 significantly increased PCa risk, with age consistently predictive. Risk signatures confirmed age as the dominant risk factor (hazard ratio 1.15 per year, 95% confidence interval 1.13-1.18) and highlighted interaction effects: younger men with cancer history had higher PCa risk, while elevated CRP with high CCI amplified risk, demonstrating strong predictive accuracy (receiver operating characteristic area under the curve: 0.82; Harrell’s C: 0.72). Categorising age and CCI further refined risk stratification.

**Conclusion:**

Although elevated CRP was associated with higher PCa risk, no clear associations were detected for other markers. Advanced risk analysis found age to be the sole predictor, indicating conventional methods may overestimate biomarker roles in PCa prediction.

## Highlights

• Advanced techniques are needed for biomarker-based cancer risk prediction.
• The aim of the study was to evaluate PCa risk using conventional and new mathematical methods.
• Conventional method was Cox, while the new approach was risk signature analysis.
• Age was the only predictor of PCa risk.
• Conventional methods may overestimate biomarker roles in cancer risk prediction.

## Introduction

Prostate cancer (PCa) is an immunogenic tumour influenced by markers of inflammation which causes dysregulation of oncogenes and tumour suppressors. A poorer prognosis for PCa has been linked to higher levels of the post-diagnosis serum inflammatory markers haptoglobin and C-reactive protein (CRP), as well as low levels of albumin.^1^^,^^2^ Research has also shown an association between elevated CRP or white blood cell (WBC) levels and an increased risk of incident PCa or PCa-related mortality.^3^, ^4^, ^5^

Previous studies in the Swedish Apolipoprotein-related MOrtality RISk (AMORIS) have produced a range of findings regarding the risk of PCa.^6^^,^^7^ While the initial study by Van Hemelrijck et al. found no association between inflammatory markers and PCa, the subsequent study by Arthur et al. explored the association by evaluating various confounders and identified some correlations. After adjusting for age, educational level, Charlson comorbidity index (CCI) and the time interval between the measurement date and diagnosis date, men with elevated levels of haptoglobin, CRP and WBC had a higher risk of developing advanced PCa. In contrast, higher albumin levels were inversely associated with an increased risk of advanced PCa.^6^^,^^7^ The results of these studies highlight the need for more advanced methodologies to better understand these associations as traditional statistical methods may not fully capture the complex interplay of factors influencing PCa risk and progression.

A prognostic risk signature defines a means of combining measured data for different risk factors (i.e. covariates) to quantitatively estimate an individuals’ risk of a given outcome, in this current work, the risk of PCa diagnosis. A systematic procedure for estimating robust risk signatures that weights those risk factors determined to have a statistically significant influence on the risk of the outcome of interest is preferred in this work.^8^^,^^9^ This method estimates a risk signature by iteratively removing the least informative covariate, identified using Cox-like time-to-event regression. To prevent overfitting and reduce estimation bias, the analysis was conducted with many randomly selected training datasets. The predictive performance of the risk signature is quantified at each iteration, for both training and (unseen) validation data, allowing the best set of covariates that together estimate outcome risk with the least risk of overfitting, to be determined: this approach ensures that the estimated risk signature is reproducible on unseen data.

Advanced methods developed to overcome limitations inherent to conventional time-to-event regression analysis are thus needed to provide robust inferences when analysing biomarker-wide cancer data, where the risks of (overfitting and therefore) unreliable inferences can be significant (e.g. when including many biomarkers).^10^, ^11^, ^12^ The validated spsSIGNATURE (spsSIG) software (Saddle Point Science Ltd., York, UK) provides algorithms that robustly estimate ‘risk signatures’ that can be used for the prediction of an individual’s personalised risk of developing the disease of interest, enabling cohort stratification.^8^^,^^9^^,^^13^

The aim of this study was to investigate the role of immune inflammatory parameters in PCa in the AMORIS cohort using two methods: Cox proportional hazards model using baseline covariates from the third time point of CRP, albumin, haptoglobin and WBC and risk signature analysis using spsSIG that incorporated repeated measures of the markers from three medical check-ups.

## Materials and methods

### Study population

The Central Automation Laboratory (CALAB) was a leading centre for analyses of blood and urine samples from health screenings and health examinations in primary health care in Sweden between 1985 and 1996. The AMORIS cohort includes >800 000 subjects with blood samples on biomarkers from these health examinations with record linkages to several Swedish national health registers and research cohorts until 2020. In this way the cohort comprises information for all subjects included on deaths after the first blood sampling, all new cancers diagnosed before and following blood sampling and all hospitalisations in Sweden after 1987.^14^ All linked data in the AMORIS cohort are anonymised and approved by the Stockholm Ethical Committee (Dnr 2010/1:7).

The AMORIS cohort is a real-world data resource and was established as part of routine annual health check-ups through occupational health care or for outpatients referred for laboratory tests; however, the time intervals between measurements were not always consistent suggesting potential diagnostic bias. To ensure the consistency and reliability of the measurements, we included only men who had three measurements which would minimise any potential bias or inconsistency in the biomarker data. To make sure that the measurements included in our study were truly from a generic medical check-up, measurements that were <9 months or >18 months apart were excluded. Moreover, to avoid reverse causation, men who were diagnosed with PCa by the time of the last measurement were excluded.

### Analysis

#### Analysis 1: Cox proportional hazard modelling

To investigate associations between the different humoral immune system markers and risk of PCa diagnosis, time-to-event analysis was conducted using multivariable Cox proportional hazard models to calculate hazard ratios (HRs) and 95% confidence intervals (CIs). Exposures were defined as baseline measurements of inflammation markers (the third time point only as this is a time-to-event analysis)—albumin, CRP, haptoglobin and WBC. The outcome was defined as a diagnosis of PCa. Follow-up time was calculated from the date of third measurement to date of PCa diagnosis or censor date. The analysis was adjusted for age, socioeconomic status, education level, CCI and other cancers.

#### Analysis 2: risk signature analysis with ‘raw’ covariates

The risk signature regression algorithm predicted PCa risk using repeated measures of CRP, albumin, haptoglobin and WBC, from three medical check-ups, included as ‘landmarked’ baseline covariates relative to the date of the third measurements. Age, socioeconomic status, education level, CCI and cancer history were also included as baseline covariates, as were covariate–covariate product terms, given the algorithm’s ability to handle more covariates than conventional methods. The outcome was defined as a diagnosis of PCa, with follow-up calculated from the date of the third measurement (i.e. the ‘landmark time’) to date of PCa diagnosis or censor date.

To avoid reverse causation, it was ensured that none of the men included had PCa at the third measurement. Correlation between the three measurements were analysed by calculating the cut-off points for each marker. CRP, haptoglobin and leukocytes were categorised based on the clinical cut-offs used in the CALAB laboratory: CRP: <10 mg/l and ≥10 mg/l; haptoglobin: <1.4 g/l and ≥1.4 g/l; WBC <10 (109 l) and ≥10 (109 l). Albumin was dichotomised as <40 g/l and ≥40 g/l instead of the clinical cut-off of 35 g/l due to the small number of participants with low albumin levels.

This cohort was randomly split into one training and one validation dataset, each of about the same size, for analysis. This was to enable the performance of the risk signature, estimated using the training data, to be quantified against the ‘unseen’ data in the validation dataset. The validated spsSIG software estimated a multivariate risk score by iteratively removing the least informative covariate and, through determination of overfitting, the set of covariates required for the optimal risk score formulae.

Missing data were addressed in the analysis using mean imputation and by including indicator variables for missingness.

#### Analysis 3: sensitivity risk score analysis with age and CCI as categorical covariates

In this analysis, age and CCI were included as categorical factors rather than in their ‘raw’ form as in analysis 1 and 2, with all other covariates encoded as they had been in analysis 2. This was done to allow easier comparison with results from analyses in the literature, where age and CCI were commonly encoded categorically. Age was categorised as follows: <40, 40-60, 60-80 and ≥80 years and CCI was categorised as 0, 1, 2 and ≥3.

#### Rationale for Cox regression and signature risk score models

Strong predictive accuracy in Cox models, especially when unusual predictors are included, often requires more than the usual 10 events per variable. The complexity of the AMORIS dataset (i.e. including both low-prevalence variables and complex patterns of missing data) adds to model complexity in Cox models (analysis 1). Addressing these missing values requires imputation methods that introduce additional variables, further increasing model complexity and raising the risk of overfitting. To address this, advanced model selection techniques were applied to analyses 2 and 3 to reduce bias and variance. By leveraging all three time points, analyses 2 and 3 make fuller use of the AMORIS dataset and aims to improve predictive accuracy over the Cox model.

## Results

Table 1 shows patient characteristics for the three time points for the four immune markers albumin (*n* = 9320), CRP (*n* = 5183), haptoglobin (*n* = 7257) and WBC (*n* = 1466).

**Table 1:** Patient characteristics of serum immunological measurements for all markers from the AMORIS cohort at three time points

Patient characteristics	Albumin (*n* = 9320)			CRP (*n* = 5183)			Haptoglobin (*n* = 7257)			White blood cells (*n* = 1466)		
Time points	1	2	3	1	2	3	1	2	3	1	2	3
Mean measurement (SD)	43.4 (2.8)	43.5 (2.8)	43.6 (2.8)	4.5 (5.1)	4.5 (4.9)	5.2 (5.2)	1.0 (0.3)	1.0 (0.3)	1.0 (0.3)	6.5 (1.7)	6.5 (1.8)	6.5 (1.8)
Mean age for all markers, years (SD)	50.8 (13.3)	52.2 (13.4)	53.6 (13.4)									
Mean follow-ups for all markers, years (SD)	17.9 (5.4)	16.5 (5.4)	15.1 (5.4)									
Age categories at time point 3, years												
<40	1834 (17.0)											
40-59	5607 (51.9)											
60-79	3074 (28.4)											
≥80	296 (2.7)											
Socioeconomic status at time point 3												
Low (%)	2674 (24.7)											
High (%)	7503 (69.4)											
Missing (%)	634 (5.9)											
Education at time point 3												
Low (%)	2319 (21.5)											
Middle (%)	4156 (38.4)											
High (%)	4004 (37.0)											
Missing (%)	332 (3.1)											
Charlson comorbidity index at time point 3												
1 (%)	727 (6.7)											
2 (%)	442 (4.1)											
3+ (%)	252 (2.3)											
Missing (%)	9390 (86.9)											
Other cancer except prostate cancer												
Yes	1572 (14.5)											
No	9239 (85.5)											

AMORIS, Apolipoprotein-related MOrtality RISk; CRP, C-reactive protein.

### Results 1—Cox proportional hazard model

Only the third time point was used for the time-to-event analysis (Table 1). Following a mean total follow-up of 15.1 years [standard deviation (SD) 5.4] at measurement 3, 678 (6.3%) men developed PCa. Table 2 shows the results for Cox proportional hazard models. Increased CRP levels were associated with a greater risk of PCa diagnosis (HR 1.05, 95% CI 1.01-1.10). Albumin, haptoglobin and WBC showed no association. Age was consistently a predictor for risk of PCa diagnosis for three immune markers including albumin (HR 1.06, 95% CI 1.03-1.09), CRP (HR 1.06, 95% CI 1.03-1.10) and haptoglobin (HR 1.07, 95% CI 1.04-1.10). Presenting with a CCI score of 2 demonstrated an increased risk of PCa diagnosis for all four immune markers: albumin (HR 2.39, 95% CI 1.39-4.10), CRP (HR 3.99, 95% CI 1.73-9.19), haptoglobin (HR 2.07, 95% CI 1.13-3.80) and WBC (HR 7.20, 95% CI 2.34-22.21). Moreover, men with a previous history of other cancer and decreased WBC showed an inverse association with PCa diagnosis (HR 0.33, 95% CI 0.11-0.95).

**Table 2:** Hazard ratios and 95% confidence intervals from Cox proportional hazard models for albumin, C-reactive protein, haptoglobin and white blood cells at time point 3

Markers Patient characteristics	Albumin, *n* = 9320 HR (95% CI)	CRP, *n* = 5183 HR (95% CI)	Haptoglobin, *n* = 7257 HR (95% CI)	White blood cells, *n* = 1466 HR (95% CI)
**MARKER**	0.96 (0.88-1.05)	1.05 (1.01-1.10)	0.97 (0.38-2.45)	0.89 (0.67-1.19)
**AGE, YEARS**	1.06 (1.03-1.09)	1.06 (1.03-1.10)	1.07 (1.04-1.10)	1.06 (1.00-1.12)
**SES**				
Low	1.00 (Ref)	1.00 (Ref)	1.00 (Ref)	1.00 (Ref)
High	1.78 (0.92-3.45)	1.57 (0.58-4.29)	2.10 (0.95-4.66)	2.10 (0.55-8.05)
**EDUCATION**				
Low	1.00 (Ref)	1.00 (Ref)	1.00 (Ref)	1.00 (Ref)
Middle	0.80 (0.42-1.52)	0.39 (0.15-0.98)	0.83 (0.39-1.76)	0.99 (0.24-4.12)
High	1.50 (0.80-2.79)	0.95 (0.41-2.22)	1.50 (0.73-3.12)	2.72 (0.69-10.73)
**CCI**				
1	1.00 (Ref)	1.00 (Ref)	1.00 (Ref)	1.00 (Ref)
2	2.39 (1.39-4.10)	3.99 (1.73-9.19)	2.07 (1.13-3.80)	7.20 (2.34-22.21)
3+	1.32 (0.66-2.64)	3.29 (1.26-8.57)	1.17 (0.52-2.61)	1.26 (0.24-6.57)
**OTHER CANCER**				
No	1.00 (Ref)	1.00 (Ref)	1.00 (Ref)	1.00 (Ref)
Yes	0.61 (0.36-1.04)	0.77 (0.38-1.57)	0.67 (0.37-1.23)	0.33 (0.11-0.95)

CCI, Charlson comorbidity index; CI, confidence interval; HR, hazard ratio; SES, socioeconomic status.

### Results 2—risk signature analysis with ‘raw’ covariates

All three measurements were available for albumin (*n* = 9320), CRP (*n* = 5183), haptoglobin (*n* = 7257) and WBC (*n* = 1466).

Out of the 5406 men with three repeated measurements of immune markers in the training set, 337 (6%) developed PCa. The risk signature algorithm identified age, included as a real-valued covariate, as the dominant risk factor (HR 1.15 per year, 95% CI 1.13-1.18). It also suggested that a history of other cancer diagnoses combined with age, and CCI combined with deranged CRP, contribute to refining the PCa risk prediction. A history of other cancer was found to be associated with elevated PCa risk for patients under 67 years and with reduced risk for older patients; elevated CRP (≥10 mg/l) increased risk, with its impact on PCa risk being amplified according to the patient’s CCI score. The estimated risk score formula provided a receiver operating characteristic (ROC) area under the curve (AUC) of 0.82 (Harrell’s C index: 0.72) after correcting for non-random covariate missingness.

### Results 3—sensitivity risk score analysis with age and CCI as categorical covariates

When age and CCI were included as categorical covariates, age was found to be the most predictive factor of the included covariates, with those in the 60- to 80-year group being at greatest risk (HR 24.8, 95% CI 10.8-57.2), followed by those in the 40- to 60-year group (HR 14.4, 95% CI 5.8-35.8); those older than 80 years had the lowest age-associated risk (HR 4.1, 95% CI 2.8-5.9); however, this may be a consequence of informative censoring due to competing risks.

Covariate interaction terms were estimated to be predictive of risk for those in the 40- to 60-year age group, with those with missing CRP data (52% of training cohort) having a reduced risk of PCa (HR 0.7, 95% CI 0.5-0.8). Moreover, a CCI of ≥3 was linked to a lower risk of developing PCa in men in the 40- to 60-year age group (HR 0.7, 95% CI 0.3-1.8).

**Figure 1:**
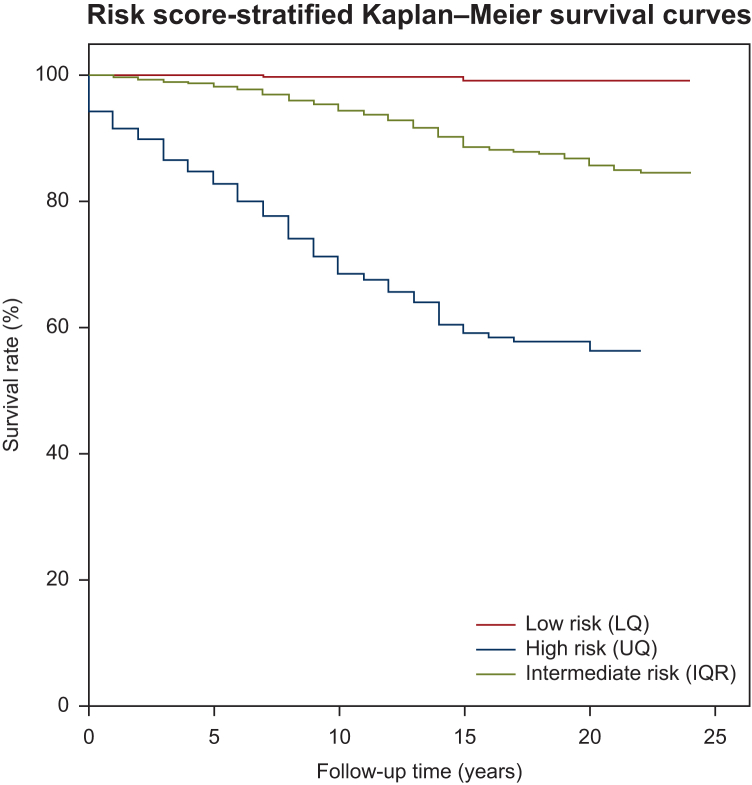
**Prostate cancer risk score-stratified Kaplan–Meier estimators following application of the risk signature determined in the sensitivity risk score analysis with age and Charlson comorbidity index as categorical covariates.** IQR, interquartile range; LQ, lower quartile; UQ, upper quartile.

Figure 1 presents the Kaplan–Meier estimators stratified by personalised risk scores, computed from the estimated risk signature, demonstrating a clear separation of patient subgroups into high-, low- and average-risk profiles for PCa. This emphasises the therapeutic significance of individualised risk stratification by demonstrating the model’s capacity to distinguish survival probability according to risk levels.

**Figure 2:**
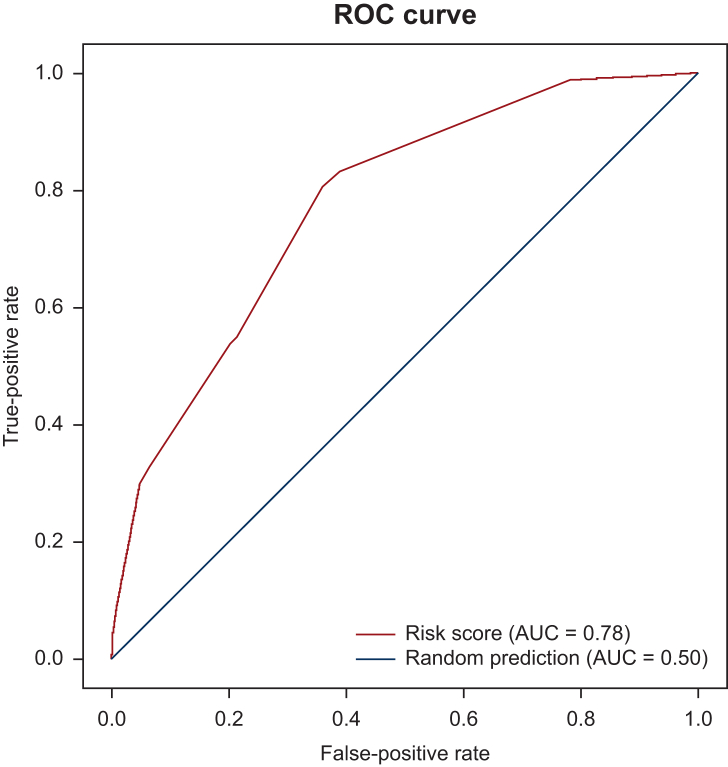
**Receiver operating characteristic (ROC) curve from the application of the risk signature determined in the sensitivity risk score analysis with age and Charlson comorbidity index as categorical covariates.** AUC, area under the curve.

Figure 2 shows the corresponding ROC curve generated using the estimated risk signature determined in the sensitivity risk score analysis with age and Charlson comorbidity index as categorical covariates, indicating how well the model predicts outcomes. The AUC was 0.78, which suggests that the risk signature was an accurate prediction tool.

## Discussion

This study confirmed that elevated CRP levels were associated with a higher PCa risk, and both conventional and advanced analyses consistently identified age as the sole reliable predictor of PCa. Age-related decline in physiological functions makes individuals more prone to conditions that cause chronic inflammation.^15^ CRP and haptoglobin are markers of inflammation, and chronic inflammation is a known cancer risk factor, contributing to the fact that the likelihood of developing PCa increases with age.

Albumin showed no significant association on its own with the risk of PCa in the current study. While age-related declines in albumin levels have been previously linked to increased PCa risk—likely due to their reflection of overall health and inflammatory status—the current results indicated a non-significant association.^16^ Further investigation is required to elucidate this link.

Known to be produced by the liver in response to infection, tissue damage and autoimmune conditions, CRP serves a range of inflammatory functions. It can potentiate immune stimulation via Fc-mediated binding to immunoglobulin receptors on effector cells and through complement binding and classical complement cascade activation. In concordance, chronic inflammation and tissue remodelling are known risk factors for and hallmarks of cancer development. Furthermore, due to their intrinsic cellular origin, several tumour types including PCa harbour autoimmune features.^17^, ^18^, ^19^, ^20^ Elevated serum CRP reflects such a broad chronic inflammation state as a predisposing feature of PCa as well as a likely temporal marker of yet undetected malignant disease. Consistent with this, men with high CRP levels (≥10 mg/l) were found to have a significantly greater chance of being diagnosed with high-risk or advanced PCa, including regional or distant metastatic disease.^7^ High CRP levels in men with PCa have also been identified as a strong indicator of poorer survival.^4^ These findings suggest that CRP levels may have the potential to serve as a valuable prognostic marker in PCa. Extensive biomarker data in the AMORIS study are limited, mostly adhering to measurements of broad inflammatory features such as CRP (however, not measured using high-sensitivity CRP). Although CRP showed a strong association with a higher risk of a PCa diagnosis in both the traditional and risk signature analyses, with a ROC AUC of 0.82, this mediator most likely operates as part of a wider inflammatory network. Therefore, it is possible that an artificial intelligence-driven collective inflammatory biomarker signature that includes CRP may be derived that can provide more accurate tools of PCa risk prediction, detection, prognosis and therapy prediction. A previous study reported correlation between CRP levels with prostate-specific antigen, and this may provide additional opportunity for the development of combinatory diagnostic and monitoring algorithms.^21^

In the risk score analysis, elevated CRP levels (≥10 mg/l) were associated with an increased risk of PCa, with this risk further increasing in men with a higher CCI score. This may be due to detection bias where more frequent medical evaluations, screenings and general health-seeking behaviours due to their multiple health conditions may explain the increase in PCa cases being diagnosed in these men.^22^ However, the association between elevated CRP and cancer risk may likely be more significant than detection bias alone. The combined effect of chronic inflammation from various sources may exacerbate the inflammatory environment in individuals with multiple comorbidities, potentially accelerating the development of PCa.^23^, ^24^, ^25^

Although elevated haptoglobin levels were associated with increased risk of PCa in the Cox model in this study, it was not highlighted as a significant marker in the risk signature analysis. Elevated haptoglobin levels have been associated with worse outcomes in non-small-cell lung cancer.^26^ While haptoglobin has not been directly linked to PCa, its role as a marker of inflammation makes it a relevant factor in understanding the broader context of cancer risk and progression, particularly in older men where inflammation may be more prevalent.

Moreover, having a history of other cancers was associated with an increased risk of PCa in men aged <67 years, while it was linked to a decreased risk in older men. However, this may be explained by detection bias where the possibility that older men with a history of other cancers may be less likely to be screened for PCa because other health conditions take precedence over PCa contributing to the lower detection rates in this age group.^22^^,^^27^ The observed decrease in detection rates for this age group may be due to competing risks, with lower PCa screening rates being a more significant factor than an actual reduction in PCa risk. The authors intend to carry out further analyses on the AMORIS data with the multi-risk latent class method.^28^ This method accounts for the influence of informative censoring due to competing risks, an issue that is well known to influence PCa studies and that may have had a bearing on the estimated HRs presented here.

This study highlights some of the limitations associated with conventional statistical methods and the need for advanced methods to effectively account for the complexities of real-world epidemiological datasets. The risk signature method applied here incorporates protection against overfitting and biases to estimate robust prognostic risk signatures, even with a large number of covariates relative to sample size. It is this that made it possible to reliably account for the influence of informative missingness (i.e. data missing not at random) in the AMORIS cohort, a particular problem in this study due to the substantial levels of missing data in the repeated measurements of inflammation markers. Ultimately, this leads to more reliable estimators throughout and should yield a reliable prognostic risk signature that can be applied to unseen data. Moreover, measurements collected over time in this study may contain variability (referred to as ‘noise’) that may not accurately reflect the true underlying patterns or relationships, which highlights the need for methods that can account for these variations.

### Conclusion

Although elevated CRP levels were associated with higher chance of PCa diagnosis in men with multiple comorbidities, no significant associations were observed for the other immune markers explored. Advanced risk signature analysis identified age as the only consistent predictor of PCa diagnosis. These findings suggest that the effects observed through conventional methods may not fully represent the true predictors of developing a disease.
